# A Rare Case of Combined Direct Retinal Involvement and Suspected Bing-Neel Syndrome in a Patient With Waldenstrom’s Macroglobulinemia

**DOI:** 10.7759/cureus.71871

**Published:** 2024-10-19

**Authors:** Muhammad Khursheed Ullah Khan Marwat, Khalid Khalil, Imad Al Assir

**Affiliations:** 1 Infectious Diseases, Hull University Teaching Hospitals NHS Trust, Hull, GBR; 2 Ophthalmology, Cairo University Teaching Hospitals, Cairo, EGY; 3 Ophthalmology, Hull University Teaching Hospitals NHS Trust, Hull, GBR; 4 Neuroradiology, Hull University Teaching Hospitals NHS Trust, Hull, GBR

**Keywords:** intraocular lymphoma, neuro radiology, radiotherapy (rt), rare clinical presentation, vitreous biopsy, vitreous haemorrhage, waldenstroms macroglobulinemia

## Abstract

A 75-year-old male with a history of Waldenström macroglobulinemia (WM), diagnosed in 2022, presented with several months of progressive blurred vision and floaters in his right eye, impairing his ability to drive, particularly at night. The ophthalmologic evaluation revealed vitreous haemorrhage and sub-retinal pigment epithelial lesions in the superonasal and inferonasal quadrants of the right eye. A pars plana vitrectomy with vitreous biopsy was performed, which was consistent with ocular involvement by WM. The patient underwent orbital radiotherapy. Shortly after completing radiotherapy, he developed acute neurological symptoms, including involuntary movements and erratic behaviour. Based on imaging and clinical presentation, the lesion was highly suspected to represent central nervous system (CNS) involvement by WM (Bing-Neel syndrome), though a tissue diagnosis could not be obtained due to the fitness of the patient. The patient was treated with rituximab and high-dose methotrexate, but after three cycles, follow-up imaging showed progressive CNS disease. Due to his declining condition, any further could not be pursued. At the time of this report, his visual acuity in the right eye was reduced to 6/60 due to silicone oil used during the vitreoretinal surgery, and further review is awaiting. This case illustrates a very rare occurrence of combined direct ocular involvement and suspected CNS infiltration in WM, highlighting the challenges of diagnosing and treating these uncommon but serious manifestations.

## Introduction

Waldenström macroglobulinemia (WM) is a rare lymphoproliferative disorder characterized by the presence of lymphoplasmacytic lymphoma and monoclonal IgM hypergammaglobulinemia. First described by Waldenström in 1944, the condition typically presents symptoms related to bone marrow infiltration and high serum viscosity [1,2]. Patients may experience oronasal bleeding, lymphadenopathy, anaemia, and thrombocytopenia [3,4]. WM predominantly affects individuals between 63 and 68 years of age, with a higher incidence in men and Caucasian populations [1,2,4]. The estimated incidence is around three cases per million per year, representing approximately 2% of all haematological cancers [2,4]. Although WM is considered indolent, the average survival rate is about five years, with some patients living for more than a decade [3,4].

The aetiology of WM remains unclear, but potential associations have been identified with autoimmune diseases, environmental factors, and chronic antigenic stimulation, such as hepatitis C virus infection [5]. Familial predisposition has also been noted, with first-degree relatives exhibiting a significantly higher risk for lymphoproliferative disorders [2,5]. The pathophysiology of WM is believed to originate from memory B lymphocytes, which undergo malignant transformation. These neoplastic B cells typically express surface IgM and IgD, indicating a block in immunoglobulin isotype switching, likely due to dysfunction in the activation-induced cytidine deaminase (AID) enzyme [2,4]. 

Clinically, WM encompasses a spectrum of symptoms related to hyperviscosity and tissue infiltration. Hyperviscosity syndrome can lead to complications, including bleeding, visual disturbances, and neurological and cardiac issues. This syndrome usually occurs when IgM levels exceed 5000 mg/dL, resulting in impaired tissue perfusion [1,2].

For treatment, asymptomatic patients are often monitored without immediate intervention, as treatment does not necessarily improve quality of life or survival. Symptomatic patients may receive therapies including alkylating agents, purine analogues, and anti-CD20 monoclonal antibodies, although WM remains incurable. Recent studies suggest potential benefits from proteasome inhibitors like bortezomib [2,4].

## Case presentation

**Figure 1:**
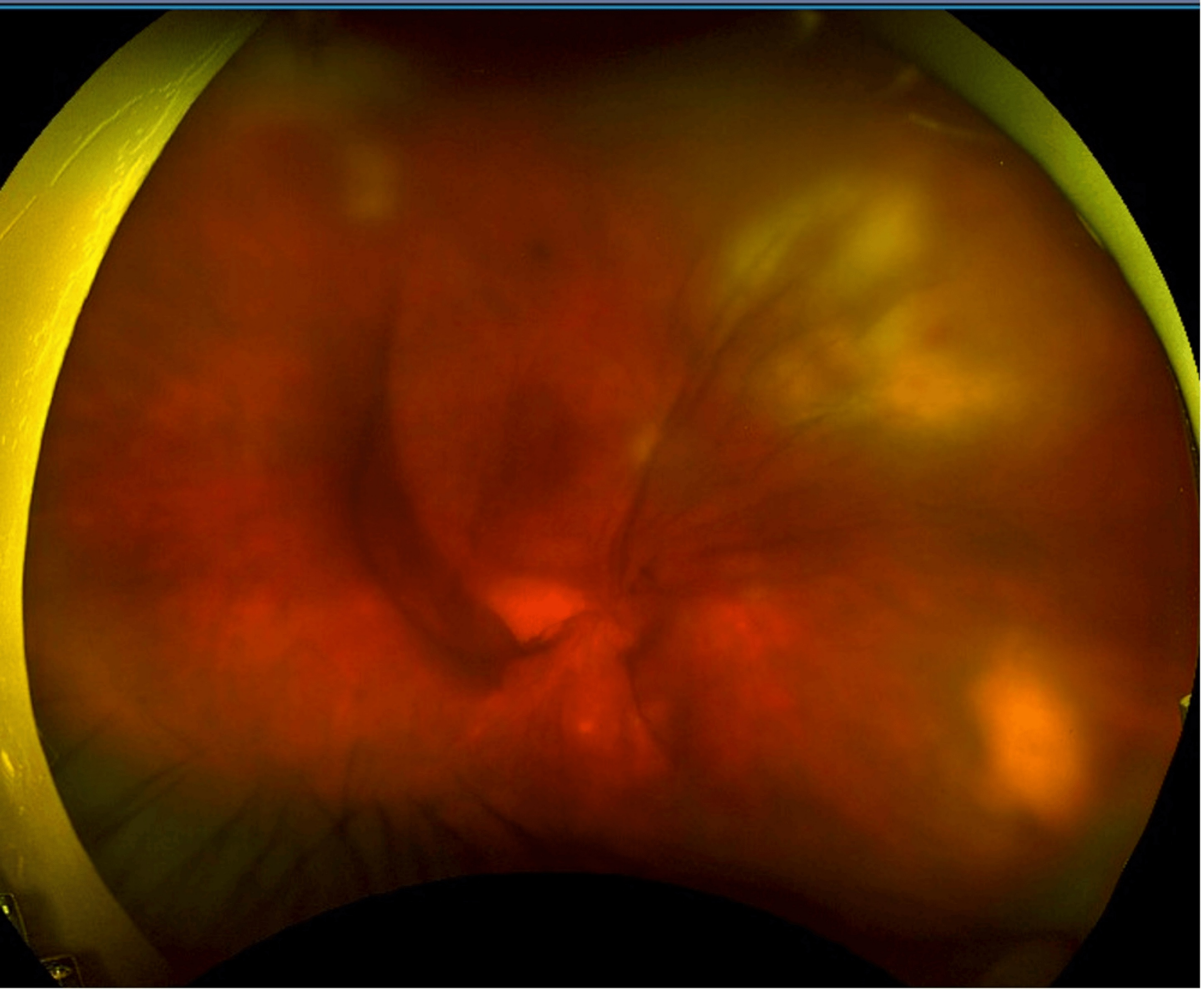
Right eye fundus image at presentation showing persistent dense vitreous haemorrhage with hazy view of superonasal fluffy ill-defined creamy white chorioretinal retinal lesion and inferotemporal raised white lesion.

This case report presents a 75-year-old man with a history of WM, a rare type of non-Hodgkin lymphoma that primarily affects lymphoplasmacytic cells. He had been under active surveillance since his diagnosis in April 2022. His recent symptoms began with complaints of blurred vision and floaters in the right eye, which had persisted for several months, impairing his ability to drive, especially at night. Upon evaluation by his optician, he was referred to an eye hospital for further evaluation. The patient denied any recent trauma, infection, or surgeries to the affected eye, but clinical examination revealed a reduction in visual acuity to 6/18 in the right eye and 6/9 in the left eye, with normal intraocular pressures of 15 mmHg bilaterally. The anterior chamber examination was unremarkable. However, fundoscopic findings showed a vitreous haemorrhage in the right eye, with a hazy view of several sub-retinal lesions in the superonasal and inferonasal quadrants (Figure 1), raising immediate concern.

**Figure 2:**
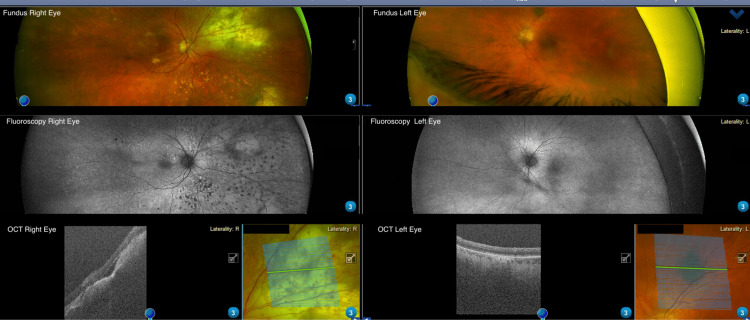
Second POD shows ophthalmic imaging studies of both eyes, with the following modalities depicted. Fundus Photographs: Right eye: retinal haemorrhages and infiltrative lesions in the periphery, particularly in the superonasal and inferotemporal region. Silicone oil in situ. Left eye: more normal appearance with accidental low-risk flat choroidal naevus along superotemporal arcade fluorescein autofluorescence. Right eye: hyper- and hypo-fluorescent spots indicate areas of RPE infiltration. Left eye: Fluorescence appears more even, with no clear signs of retinal abnormalities. OCT right eye: subretinal fluid and sub RPE infiltration. Left eye: the OCT scan over choroidal naevus with no subretinal fluid with flat choroidal lesion. POD: post-operative day; OCT: optical coherence tomography; RPE: retinal pigment epithelium.

Given the clinical suspicion of chorioretinitis and the possibility of intraocular lymphoma, the patient underwent a right eye pars plana vitrectomy and a vitreous biopsy to obtain material for viral PCR and haematological cytology analysis. Intraoperative findings were highly suspicious of viral retinitis with a risk of retinal necrosis and retinal breaks. To mitigate the risk of rhegmatogenous retinal detachment, the surgeon opted to inject silicone oil as tamponade and started the patient on systemic antiviral therapy with oral Valganciclovir. Two days later, the patient received an intravitreal injection of Foscarnet to further manage the suspected viral retinitis while awaiting the results of the biopsy (Figure 2).

**Figure 3:**
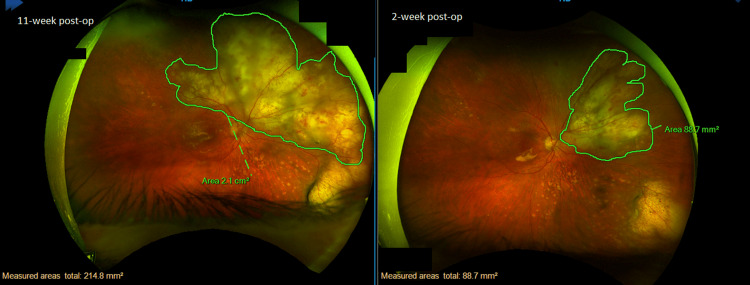
Right eye superonasal retinal infiltrate lesion almost doubled in size in the span of 9 weeks. Size: from 88.7 mm^2^ to 2.1 cm^2^.

**Figure 4:**
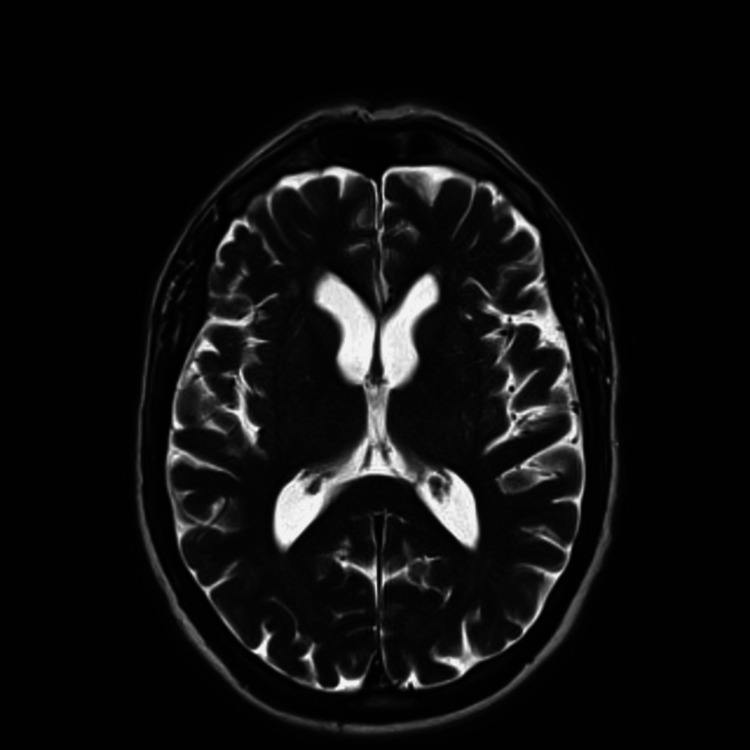
T2 weighted axial image on MRI does not show any lesion in the thalamus.

Over the course of the next nine weeks, while the patient was undergoing further investigations and multidisciplinary team (MDT) discussions, involving ocular-oncology, haematology, and neurology teams, the right eye's superonasal retinal infiltrate nearly doubled in size, expanding from 88.7 mm² to 2.1 cm² (Figure 3), signifying rapid disease progression. Analysis of undiluted vitreous samples and vitreous washings revealed the presence of neoplastic B cells, indicating vitreous involvement by the patient’s known B-cell lymphoproliferative disorder (B-LPD), consistent with WM. Flow cytometry confirmed that 45% of the leukocytes in the sample were neoplastic B cells. However, the sample was inadequate for testing mutations in MYD88 and CXCR4, which are frequently associated with WM. Following discussion in an MDT meeting, it was concluded that WM indeed had vitreous involvement. The team reached a consensus to administer radiotherapy to both orbits, with the patient receiving a total dose of 24 Grey (Gy) in 12 fractions over two weeks. During this period, an MRI of the head revealed no active brain disease (Figure 4).

**Figure 5:**
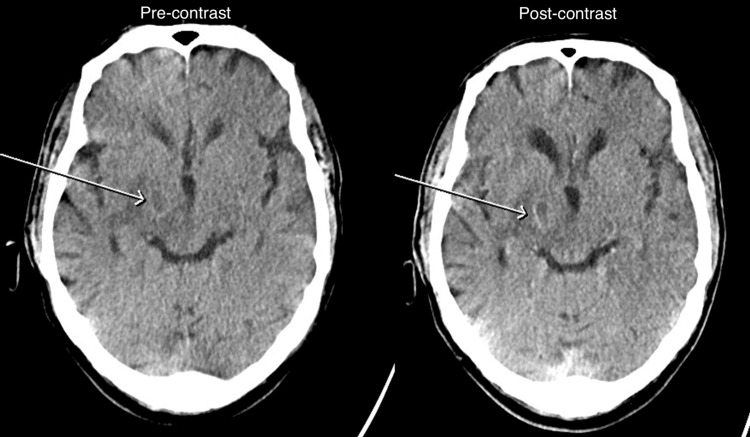
CT scan: Small hypodensity in the right thalamus (arrow) with oedema and mild peri-lesional contrast enhancement. Pre-contrast image (left) versus post-contrast image (right).

Following orbital radiotherapy, the patient developed acute neurological symptoms, including involuntary movements and erratic behavior. An urgent CT scan of the head with contrast revealed a 1.5 cm hypodense lesion with peripheral contrast enhancement in the right thalamus (Figure 5). A follow-up MRI head with contrast showed a ring-enhancing lesion with marked diffusion restriction along its enhancing wall, involving the right thalamus, the posterior limb of the right internal capsule, and the right cerebral peduncle. These findings were highly concerning for central nervous system (CNS) involvement of WM, specifically Bing-Neel syndrome, a rare but serious complication where WM infiltrates the central nervous system. Serological tests ruled out toxoplasmosis, and the MDT agreed that the findings represented the CNS spread of WM. The patient was started on Rituximab and high-dose methotrexate chemotherapy.

**Figure 6:**
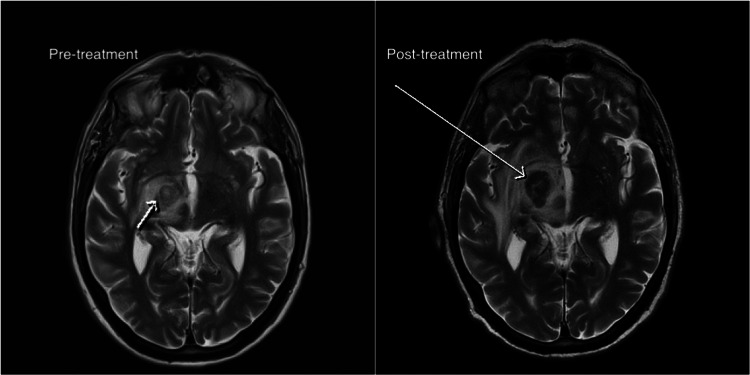
Pre and post-treatment axial T2-weighted images (10 weeks apart) showing increase in size from 12 mm (small arrow) to 25 mm (large arrow) of the right thalamic lesion with surrounding vasogenic oedema and mass effect over the third ventricle.

**Figure 7:**
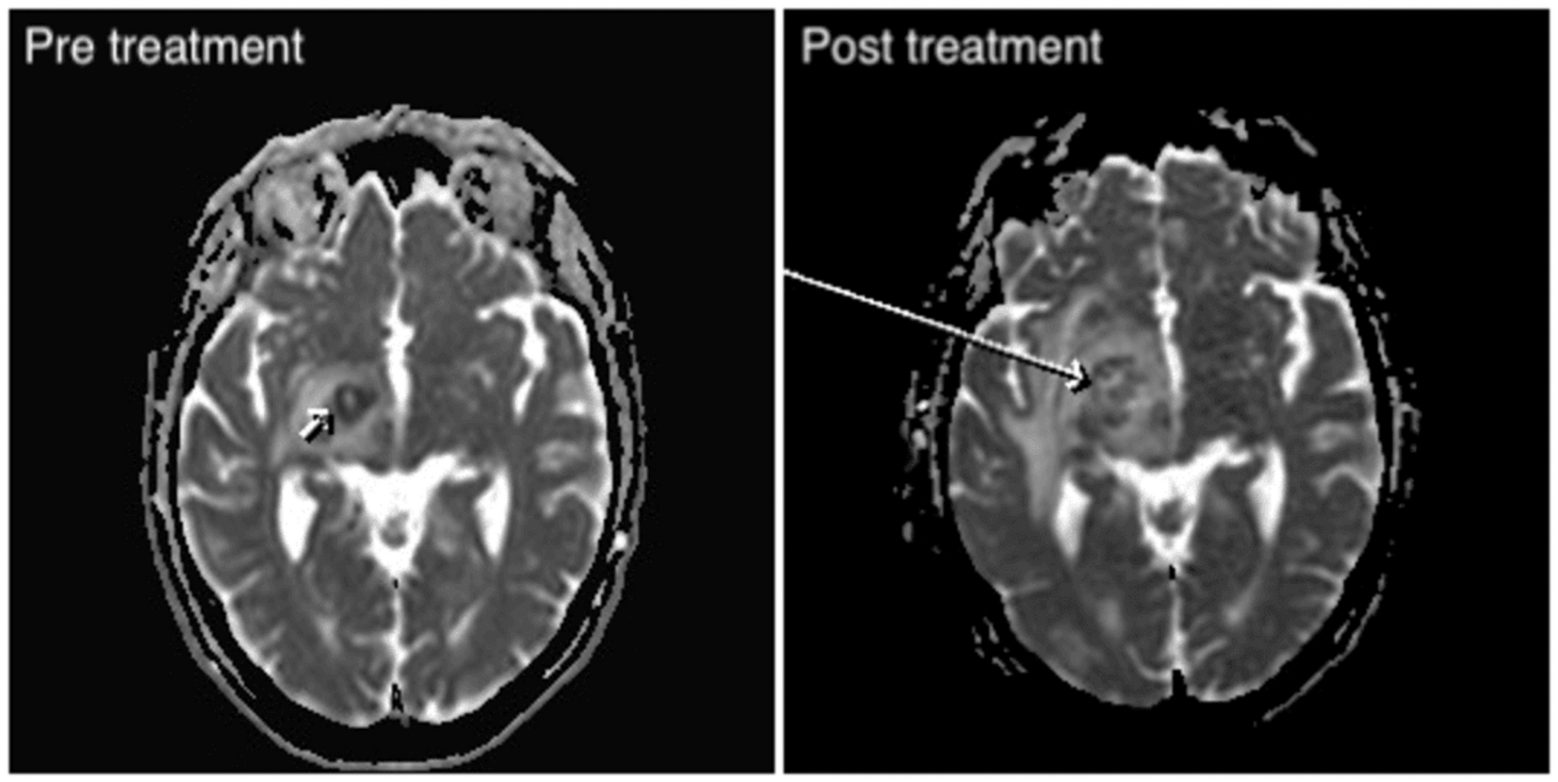
Comparison of pre-treatment (left) and post-treatment (right) axial MRI images showing peripheric signal loss on ADC sequence (small and large arrow). ADC: apparent diffusion co-efficient.

Unfortunately, after completing three cycles of chemotherapy, a follow-up MRI of the head showed progressive CNS disease, indicating that the treatment had not been successful (Figures 6-7). Further MDT discussions concluded that the patient was no longer fit for further investigations of the CNS lesion, and it was decided that no additional chemotherapy would be administered due to the lack of treatment response.

At the time of writing this case report, the patient's visual acuity was 6/60 in the right eye, largely due to the presence of silicone oil in the vitreous cavity, which had been used as a tamponade during the earlier vitrectomy and cataract progression. He was awaiting a review with vitreoretinal surgeons to determine the next steps in his ophthalmologic care, which might include the removal of the silicone oil and a cataract surgery.

## Discussion

Pathophysiology

The pathogenesis of WM involves a combination of genetic, epigenetic, and environmental factors. The hallmark mutation in WM is in the MYD88 gene, specifically the L265P mutation, found in over 90% of patients [3]. This MYD88 L265P mutation leads to constitutive activation of nuclear factor kappa B (NF-κB) signalling, promoting cell survival and proliferation. Additionally, mutations in the CXCR4 gene, present in about 30-40% of WM cases, further enhance cell proliferation and confer resistance to certain therapies [6]. Other molecular abnormalities in WM include deletions and mutations in genes involved in DNA repair, cell cycle regulation, and apoptosis, such as TP53 and ARID1A. These genetic alterations contribute to the clonal expansion of malignant B-cells and their resistance to apoptosis [7].

Prevalence

WM is a rare disorder, with an incidence rate of approximately three to four cases per million people per year in the United States. It predominantly affects older adults, with a median age at diagnosis of 70 years [8]. The disease shows a slight male predominance and is more common in Caucasian populations compared to other ethnic groups [9].

Diagnosis

The diagnosis of WM is established through a combination of clinical, laboratory, and histopathological findings. Key diagnostic criteria include the presence of a serum IgM monoclonal protein, bone marrow infiltration by lymphoplasmacytic cells, and the absence of other lymphoproliferative disorders. A bone marrow biopsy typically reveals infiltration by small lymphocytes and plasma cells [10]. Laboratory studies often show elevated serum IgM levels. Imaging studies, such as CT or PET scans, may be used to assess organomegaly or lymphadenopathy [11]. A vitreous biopsy is essential for diagnosing vitreoretinal lymphoma (VRL). This sample can be evaluated using a variety of tests, including cytology, immunohistochemistry, mutation analysis, polymerase chain reaction (PCR) for monoclonal IgH and T-cell receptor (TCR) gene rearrangements, flow cytometry, and IL-10 and IL-6 analysis [11]. Although no specific studies have been conducted on the optimal diagnostic test for detecting WM in a vitreous biopsy, a meta-analysis by Sehgal et al. concluded that the MyD88 mutation has a sensitivity of 69-88% for diagnosing VRL. The detection of a MyD88 mutation is a key indicator for diagnosing B-cell lymphomas, including primary vitreoretinal lymphoma (PVRL), central nervous system diffuse large B-cell lymphoma (CNS DLBCL), or other forms of B-cell lymphoma [12].

Bing-Neel syndrome can be divided into two main forms: diffuse and tumoral. The diffuse form is characterized by lymphoid cell infiltration in the leptomeningeal and perivascular spaces, typically seen as contrast enhancement or thickening of the meningeal sheaths. This is most effectively visualized with T1-weighted imaging (T1 WI) following gadolinium administration. On the other hand, the tumoral form can present as either a single lesion or multiple lesions, usually located in the deep subcortical regions of the brain. These lesions are best detected on T1 WI, FLAIR sequences, and T1 WI after gadolinium contrast [13].

Management

The management of WM is tailored to the patient's symptoms and disease progression. Asymptomatic patients are often monitored without immediate treatment. For symptomatic patients, therapeutic options include chemotherapy (alkylating agents like chlorambucil, cyclophosphamide, and bendamustine) and monoclonal antibodies like Rituximab, often in combination with chemotherapy [4,9]. Targeted therapies, such as Ibrutinib (a Bruton's tyrosine kinase (BTK) inhibitor), have shown efficacy in treating WM, particularly in patients with the MYD88 L265P mutation [14]. Moreover, autologous stem cell transplantation may be considered for selected patients with refractory or relapsed disease [15].

Prognosis

WM is generally considered an indolent disease with a median survival of 5-10 years. Prognostic factors include age, haemoglobin level, platelet count, beta-2 microglobulin level, and serum monoclonal protein concentration. The International Prognostic Scoring System for WM (IPSSWM) is often used to stratify patients into different risk categories [16].

Complications

Complications of WM include hyperviscosity syndrome, which can lead to neurological symptoms, bleeding, and visual disturbances. Peripheral neuropathy is another common complication, often related to the deposition of IgM or its interaction with myelin-associated glycoprotein [17]. Renal dysfunction, cryoglobulinemia, and amyloidosis are also potential complications [18]. Moreover, some rare but significant complications include direct ocular and CNS involvement (Bing-Neel syndrome) [19].

Ocular symptoms in WM

Ocular symptoms of vision changes in WM are seen in 30% to 40% of patients and are due to hyperviscosity syndrome. Retinal findings include retinal vein engorgement with vessel tortuosity, retinal haemorrhages, microaneurysms, and optic disc oedema. Decreased blood flow occurs with an increased concentration of macroglobulins and blood viscosity. The rise in intravascular pressure causes retinal outflow venous obstruction, leading to retinal haemorrhages, retinal vascular tortuosity, venous dilation, and macular oedema. Cases of direct ocular spread of WM have been documented, where the disease infiltrates the retina, choroid, or optic nerve. While extremely rare, it has been reported in several case reports [20-22].

## Conclusions

WM is a rare and complex haematologic malignancy with distinct clinical and molecular features. Recent advances in understanding its genetic basis, particularly through identifying MYD88 and CXCR4 mutations, have paved the way for more targeted therapeutic approaches. Although typically indolent, WM can cause considerable morbidity due to its various complications, including rare but significant ocular and CNS involvement, which often present unique diagnostic challenges. Ongoing research remains crucial to further enhance the diagnosis, treatment, and overall prognosis of this difficult-to-manage disease.
